# Testis-Specific Bb8 Is Essential in the Development of Spermatid Mitochondria

**DOI:** 10.1371/journal.pone.0161289

**Published:** 2016-08-16

**Authors:** Viktor Vedelek, Barbara Laurinyecz, Attila L. Kovács, Gábor Juhász, Rita Sinka

**Affiliations:** 1 Department of Genetics, University of Szeged, Szeged, Hungary; 2 Department of Anatomy, Cell and Developmental Biology, Eötvös Loránd University, Budapest, Hungary; National Cheng Kung University, TAIWAN

## Abstract

Mitochondria are essential organelles of developing spermatids in Drosophila, which undergo dramatic changes in size and shape after meiotic division, where mitochondria localized in the cytoplasm, migrate near the nucleus, aggregate, fuse and create the Nebenkern. During spermatid elongation the two similar mitochondrial derivatives of the Nebenkern start to elongate parallel to the axoneme. One of the elongated mitochondrial derivatives starts to lose volume and becomes the minor mitochondrial derivative, while the other one accumulates paracrystalline and becomes the major mitochondrial derivative. Proteins and intracellular environment that are responsible for cyst elongation and paracrystalline formation in the major mitochondrial derivative need to be identified. In this work we investigate the function of the testis specific *big bubble 8* (*bb8)* gene during spermatogenesis. We show that a *Minos* element insertion in *bb8* gene, a predicted glutamate dehydrogenase, causes recessive male sterility. We demonstrate *bb8* mRNA enrichment in spermatids and the mitochondrial localisation of Bb8 protein during spermatogenesis. We report that megamitochondria develop in the homozygous mutant testes, in elongating spermatids. Ultrastructural analysis of the cross section of elongated spermatids shows enlarged mitochondria and the production of paracrystalline in both major and minor mitochondrial derivatives. Our results suggest that the Bb8 protein and presumably glutamate metabolism has a crucial role in the normal development and establishment of the identity of the mitochondrial derivatives during spermatid elongation.

## Introduction

Insects have some of the longest sperm in the animal kingdom, although they contain the same components as the mammalian sperm, namely compact nucleus, acrosome, axoneme, mitochondria and plasma membrane. Mitochondria are essential organelles of both somatic and germ cells. In addition to providing energy for the cells through oxidative phosphorylation, mitochondria play important roles in signalling, differentiation and cell death. Mitochondria change shape and localization during *Drosophila* spermatogenesis [1]. During early stages of spermatogenesis, the number and appearance of mitochondria are similar to the mitochondria of somatic cells. They are distinct and dispersed through the cytoplasm. In mammals, after meiosis, a sheath of ring-shaped mitochondria organises around the axoneme in the mid-piece of the mature sperm cells [2]. In many insects, including *Drosophila melanogaster*, after completion of meiosis II, mitochondria form the so-called Nebenkern, which exists in two halves, layered on each other. After the unwinding of the two mitochondrial derivatives, they elongate, differentiate into major and minor derivatives and run along the entire length of the giant tail of the spermatid [1]. Mitochondrial derivatives and the cytoplasmic microtubules have interdependent roles in spermatid elongation, which results in a 185-fold increase in the length of the spermatids [3]. Formation and orientation of the major and minor mitochondrial derivatives and their angular position—in relation to the axoneme—are precisely defined [1]. The mitochondrial derivatives behave differently during elongation of the spermatid: the major derivative is filled with electron-dense paracrystalline, while the minor derivative has a reduced volume without paracrystalline accumulation [1]. Similarly to nuclear elongation and condensation, the differentiation of mitochondrial derivatives is synchronized in the elongating cyst.

Many ubiquitously expressed genes have a paralogue with a testis-specific expression pattern, suggesting that testis-specific duplicates have a distinct or specialized function from their parental counterparts during spermatogenesis [4]. Gene duplication could allow the testis-specific counterpart to become optimized for a testis-specific function, without compromising the ubiquitous function. Through screening of male sterile collections in *Drosophila*, it was possible to identify the function of several testis-specific genes, such as the role of Sneaky as an acrosomal component, protamines as part of the compact nucleosome of the sperm nucleus, betaTub85D, as a testis-specific tubulin, and the mitochondrial cytochrome-C-d [5][6][7]. Increasing numbers of male sterile mutants with mitochondrial defects suggest a fundamental role for mitochondria during spermatogenesis [3][8][9][10].

Mitochondrial glutamate dehydrogenases (GLUDs) are central catalytic enzymes of the reversible reaction of L-glutamate to alpha-ketoglutarate using NAD(P)+ and/or NAD(P)H+ as coenzymes [11]. GLUDs are evolutionarily conserved, localized mainly to the mitochondria, but can also be found in the cytoplasm, endoplasmic reticulum and nucleus [12]. Glutamate, a non-essential amino acid, serves as a key molecule in several biological processes, such as the citric acid cycle and neurotransmission, and it is a precursor of GABA and glutathione [13].

Here we report a male sterile allele of the testis-specific *bb8* and characterize it during the development of spermatids. We describe its expression pattern during spermatogenesis and show that *bb8* mRNA level is high in post-meiotic stages. We demonstrate that the protein is localized in the mitochondria and show that the lack of Bb8 results in the formation of megamitochondria and abnormal distribution of paracrystalline, which appears in both mitochondrial derivatives of the spermatids. Together, these findings suggest that that Bb8 is required for defining the identity of mitochondrial derivatives and, therefore, for the normal development of the spermatids.

## Results

### *Minos* element insertion in *bb8* results in male sterility

**Fig 1:**
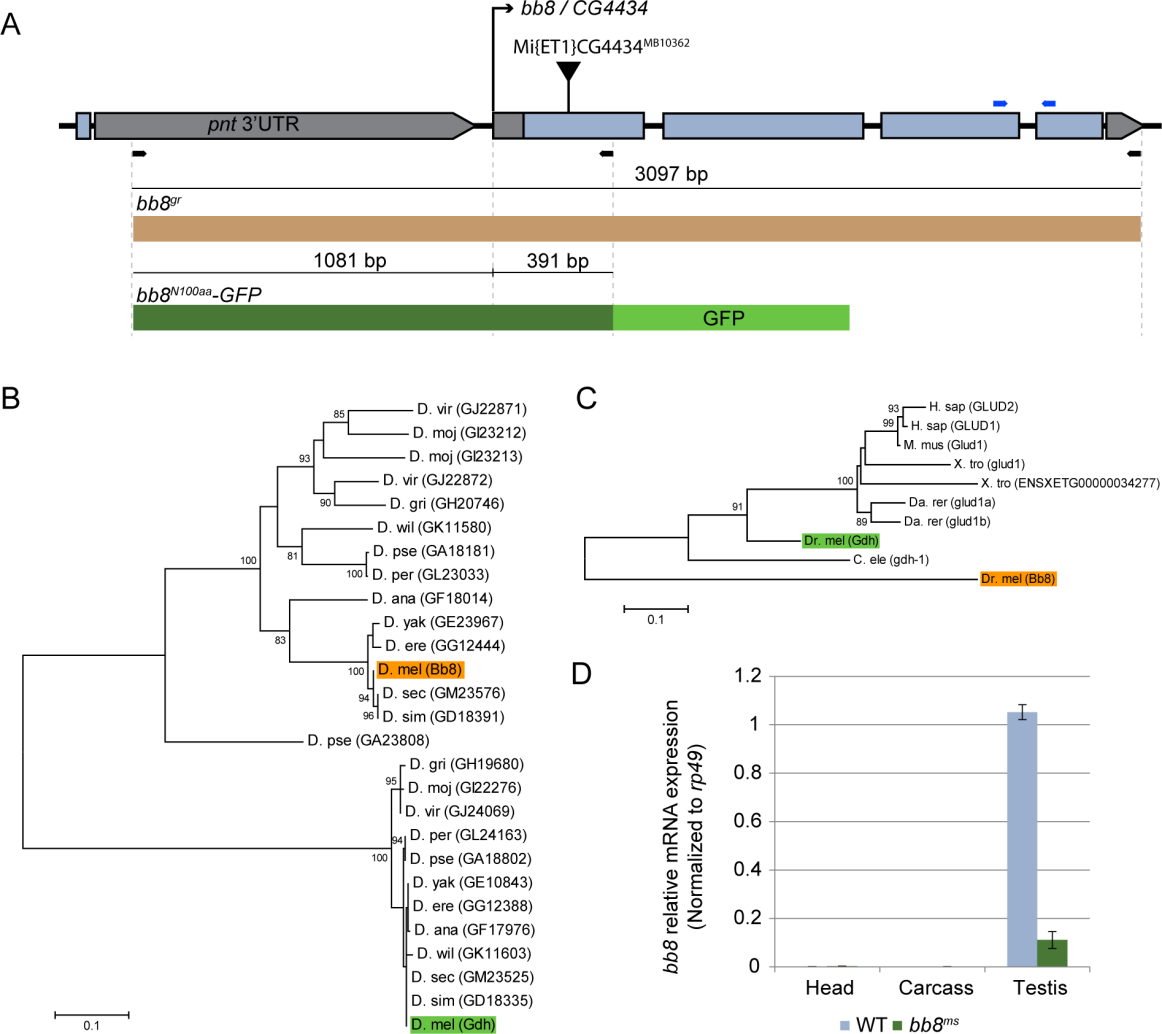
Bb8 is a testis-specific glutamate dehydrogenase. (A) Genomic organization of the *bb8* locus and the *Mi{ET1}CG4434*^*MB10362*^ transposon insertion in the first exon of *bb8*. Thick grey bars represent UTR sequences, thick blue bars the coding sequences and thin black bars the intronic and non-coding regions. Schematic shows the genomic rescue construct (*bb8*^*gr*^) (brown bar) and the genomic region (dark green bar) used to make the GFP reporter construct (*bb8*^*N100aa*^). Arrows indicate primer pairs used in the cloning of transgenes (black) and in the Q-RT-PCR (blue). (B-C) Evolutionary conservation showed of Bb8 in *Drosophila* species (B) and higher organism (C). Maximum-likelihood (B, C) method was used to construct the bootstrap consensus protein phylogeny. Bootstrap values are indicated next to the relevant nodes. (D) The expression of *bb8* mRNA is restricted to testis and show a strong reduction in *bb8*^*ms*^ mutant testis samples. Relative *bb8* expression was measured by Q-RT-PCR from wild type (WT) and *bb8*^*ms*^ mutant, from isolated head, carcass and testis samples using *bb8* and *rp49* specific primers. Measurements were made in triplicates, *rp49* was used as reference.

In an effort to identify novel genes involved in spermatogenesis, we screened the uncharacterized *Minos* transposon insertion lines from Bloomington Stock Center for male sterility and identified a line *CG4434*^*MB10362*^ (*bb8*^*ms*^) with a 100% male sterile phenotype (Fig 1A, S1A Fig). Homozygous *bb8*^*ms*^ females are fertile. Fertility was also tested on hemizygous males which carried *bb8*^*ms*^ in trans to overlapping deficiencies (*Df(3R)BSC619* and *Df(3R)Exel9012*) and found 100% male sterility (S1A Fig). *Minos* element was inserted in the first exon of *bb8* gene (Fig 1A).

The precise excision of the *Minos* element in *bb8*^*ms*^ by transposon remobilization restored wild type fertility (S1A Fig, S2C, S2G, S2K, S2O and S2S Fig) [14]. To rescue the phenotype, we made a construct by cloning the full genomic region and 1081 bp 5’ upstream of the genomic region of *bb8* into fly transformation vector (Fig 1A). The sterile phenotype of homozygous and hemizygous *bb8*^*ms*^ mutants was completely rescued by the introduction of a wild type *bb8* transgene (*bb8*^*gr*^) (S1A Fig, S2D, S2H, S2L, S2P and S2T Fig). These data further confirmed that the observed mutant phenotype is due to the disruption of *bb8* gene.

### *bb8* encodes a putative glutamate dehydrogenase

Based on sequence homology *Drosophila melanogaster* has two glutamate dehydrogenase genes, *Gdh* and *bb8* (Fig 1B). Phylogenetic analysis of protein sequences shows that Gdh is more similar to human and mouse glutamate dehydrogenases (Fig 1C). Bb8 is in a distinct phylogenetic line, but it is conserved in Drosophilidae (Fig 1B). According to the FlyAtlas database, *bb8* mRNA is highly expressed in the testes [15]. To verify the testis-specific expression of *bb8*, we carried out quantitative RT-PCR from testis, carcass, and head of wild type flies (Fig 1D). We measured *Gdh* expression parallel with *bb8*. We found that *bb8* was exclusively expressed in testis, while *Gdh* expression was detectable in the testis, head and carcass as well (Fig 1D and S1B Fig). We measured the *bb8* mRNA level in the testes of heterozygotes and homozygotes of *bb8*^*ms*^ mutants by quantitative RT-PCR, and we found an 80% reduction of gene expression in the *bb8*^*ms*^ homozygotes, demonstrating that *bb8*^*ms*^ is a strong hypomorphic allele (Fig 1D and S1C Fig).

### Expression of *bb8* and mitochondrial localization of Bb8 during spermatogenesis

**Fig 2:**
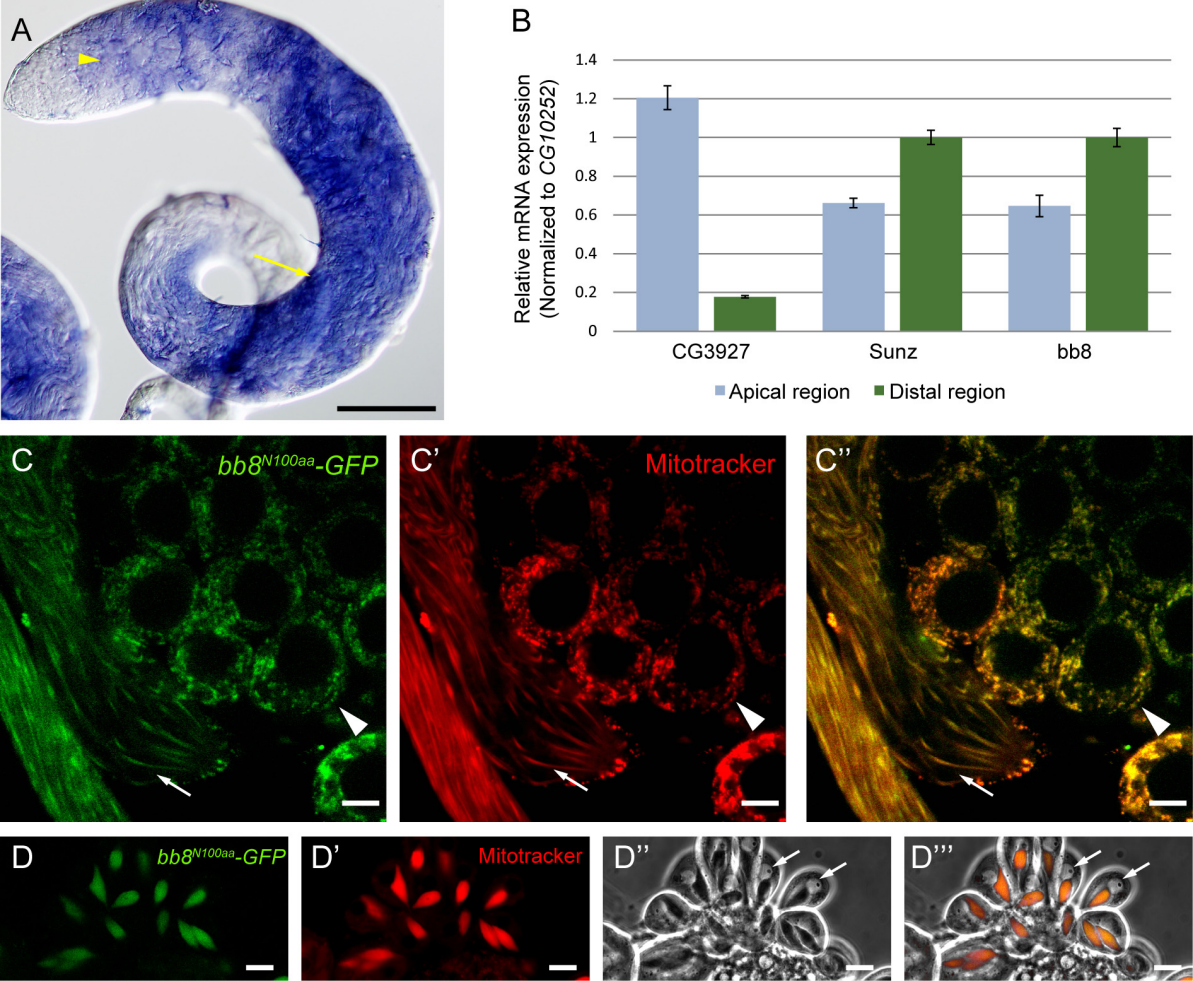
*bb8* expression in testis. (A) *In situ* hybridization with the antisense *bb8* probe shows the expression of *bb8* from the 8 cells stage (arrowhead) and RNA is present in the meiotic cysts (arrow) in WT testis. Scale bar: 200 μm. (B) Relative mRNA expression of *CG3927*, *sunz* and *bb8* were measured from dissected WT testis tip and post-meiotic region with Q-RT-PCR. mRNA expression was normalized to *CG10252*. Measurements were made in triplicates. (C, D) Expression of genomic reporter construct, *bb8*^*N100aa*^*-GFP* shows mitochondrial localization by staining with Mitotracker in spermatocytes (arrowhead), elongating spermatids (C, C’, C”) (arrow) and in round spermatids (D, D’, D”‘). Nuclei (arrow) are visualized by phase contrast microscopy (D”, D”‘). Scale bar: 10 μm.

To analyze the testis-specific distribution of the transcript, we performed *in situ* hybridization using an antisense DIG-labelled probe. The *bb8* mRNA expression pattern was found to be similar to genes expressed in late elongated cysts such as *CG10252* or *don juan* [16] [17]. *bb8* mRNA starts to express in the primary spermatocytes and shows strong RNA enrichment in the meiotic and early post-meiotic stages (Fig 2A).

To confirm the post-meiotic enrichment of the *bb8* transcript, we performed quantitative RT-PCR from the apical region and the distal region of the wild type testis (S1E Fig) [18]. As a control we used *CG3927*, which is expressed mainly in primary spermatocytes, and *sunz*, which is enriched in the elongated cysts [16]. Relative mRNA expression level was measured by quantitative RT-PCR and normalized to *CG10252* [16]. Similarly to the *sunz* transcript, *bb8* mRNA level was low in the tip region of the testis and increased in the samples from elongated cysts (Fig 2B). In humans, GLUDs localize mainly to the mitochondria and it has been shown that the N-terminal part of the protein is responsible for mitochondrial targeting [19]. We made a reporter construct, where 1081 bp of the 5’ genomic region and the first 391 bp -including the first 100 amino acids- of Bb8 were fused with GFP (*bb8*^*N100aa*^*-GFP*) in a fly transformation vector (Fig 1A and Fig 2C and 2D). We detected the expression of the reporter construct from the 8-cell stage of spermatogenesis, confirming the *in situ* hybridization data. GFP signal was localized to the mitochondria in spermatocytes and maintained its localization after meiosis in the Nebenkern and in the elongating spermatids, where GFP signals decorate the elongating tail (Fig 2C and 2D). These results confirm that Bb8 is expressed in the germ line from the 8-cell stage onward and the first 100 amino acids of the protein are sufficient for mitochondrial targeting.

### Late spermatogenesis defect leads to male sterility in *bb8*^*ms*^ mutant

**Fig 3:**
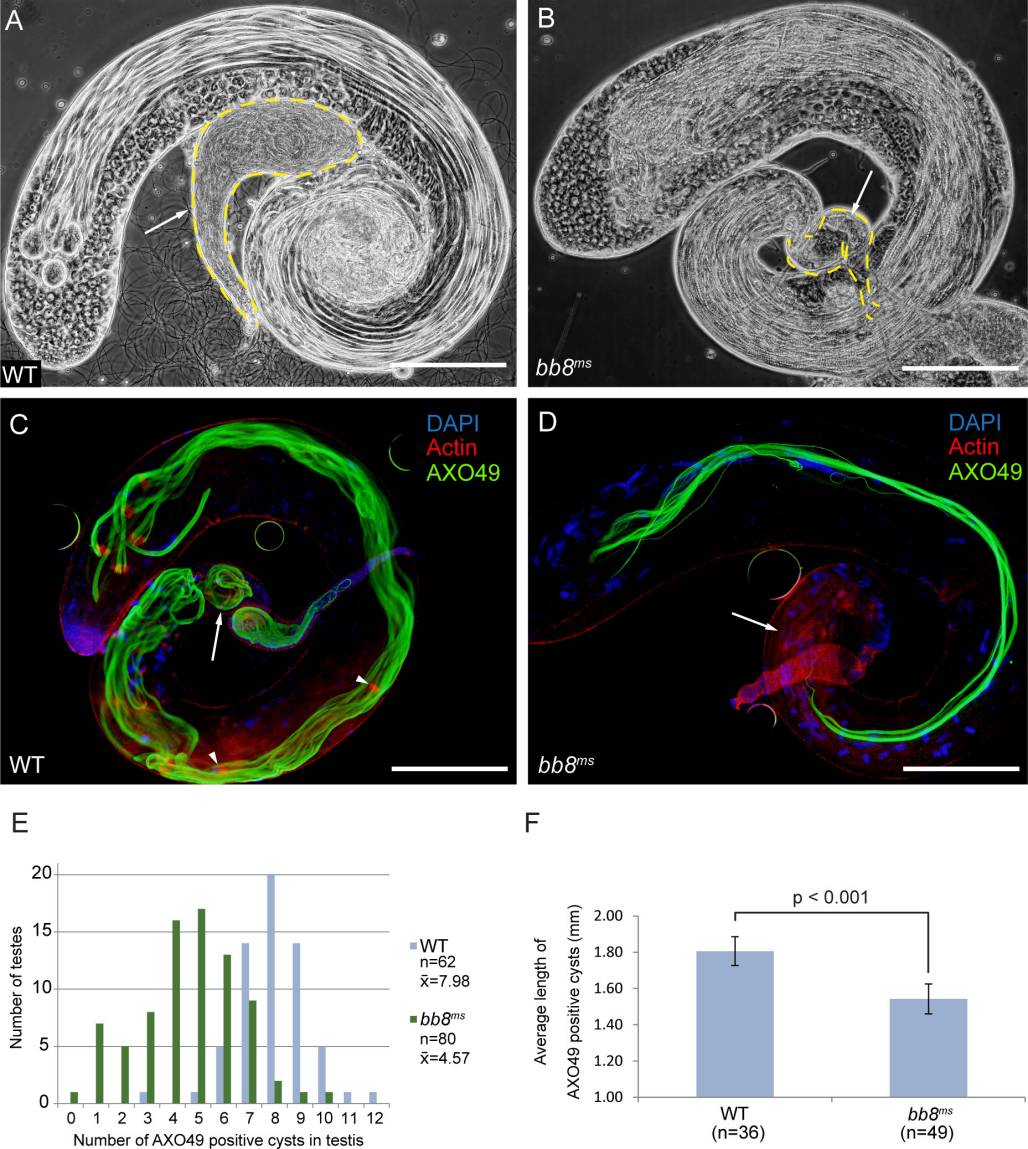
Failure in late spermatogenesis caused male sterility in *bb8*^*ms*^. (A) In WT testis the cysts in different stages and the seminal vesicles with matured sperms (arrow) are distinguishable by phase contrast microscopy. (B) In *bb8*^*ms*^ mutant testis, the early stages of spermatogenesis are normal and elongated cysts are detected, but the seminal vesicle (arrow) is empty, without mature sperms. (C, D) Elongation and individualization is disrupted in *bb8*^*ms*^ mutant testis. (C) Elongated cysts in WT testis contain actin rich individualization complexes, labelled by Texas Red-X phalloidin staining (red). Elongated cysts and coiled spermatids (arrow) are labelled by polyglycylated axonemal tubulin-specific AXO 49 staining (green). (D) In *bb8*^*ms*^ mutant testis there are no individualization complexes, there are less polyglycylated cysts per testis and there are no coiled spermatids (arrow). Nuclei stained by DAPI. Scale bars: 200 μm. (E) In WT (blue) testis there are an average 7.98 AXO 49 positive cysts per testis. In *bb8*^*m*^ (green) mutants the number of AXO 49 positive cysts is decreased, to an average of 4.57 per testis. (F) The average length of AXO 49 positive cysts are shown in WT (n = 36) and in *bb8*^*ms*^ mutants (n = 49). Error bars indicate mean ± s.e.m. Statistical significance was determined by Student’s t-test (p<0.001).

In order to understand the function of Bb8 during spermatogenesis, we tested the morphology of *bb8*^*ms*^ mutant testes. We found that seminal vesicles were empty, without mature sperm (Fig 3A and 3B and S1Q and S1R Fig).

We analysed the different developmental stages of spermatogenesis and found that the early phases were normal in mutant (S2A and S2B Fig). Meiotic cells developed properly, but we could detect different types of abnormalities in the elongated spermatid bundles of the *bb8*^*ms*^ mutant testes (S2E and S2F Fig). Nuclei of the wild type spermatids are needle-shaped after nuclear elongation (S2I Fig and S3A Fig). Even though nuclei of spermatids from *bb8*^*ms*^ were needle-shaped, they were often scattered over the apical area of the bundles (S2J Fig and S3B Fig). Axonemal tubulins of the elongated spermatids are polyglycylated (AXO 49), a modification which identifies the advanced elongated spermatids and therefore elongated cysts [20]. To analyze the elongated spermatids, we stained wild type and *bb8*^*ms*^ mutant testes with AXO 49 antibodies, then counted the number and measured the lengths of AXO 49 positive cysts. There is an average of 7.98 AXO 49 positive cysts in the testis of a 2 days old wild type male (Fig 3C and 3D). In the *bb8*^*ms*^ mutant there are 4.57 AXO 49 positive cysts per testis (Fig 3E). We measured the length of the AXO 49 positive cysts and found that the *bb8*^*ms*^ mutant has significantly shorter cysts (1.55 mm) compared to the control (1.8 mm) (Fig 3F). Individualization of the cyst containing the elongated spermatids starts after completion of elongation [21]. To visualize spermatid individualization, we used phalloidin staining, which labels the cone-shaped actin-rich structures of the individualization complex (IC) (Fig 3C and 3D, S2M and S2N Fig, S3A’ and S3B’ Fig) [22]. Actin cones started to form around the elongated nuclei of *bb8*^*ms*^ cysts, but they were not able to form a cone-shaped structure and they became dispersed in the cysts (S1N Fig, S3B’ Fig and S3C Fig). These results suggest that proper elongation of spermatids is affected in *bb8*^*ms*^ mutants, and the lack of individualization could be the consequence of impaired elongation.

### *bb8*^*ms*^ mutants exhibit mitochondrial morphology defects and megamitochondria formation

**Fig 4:**
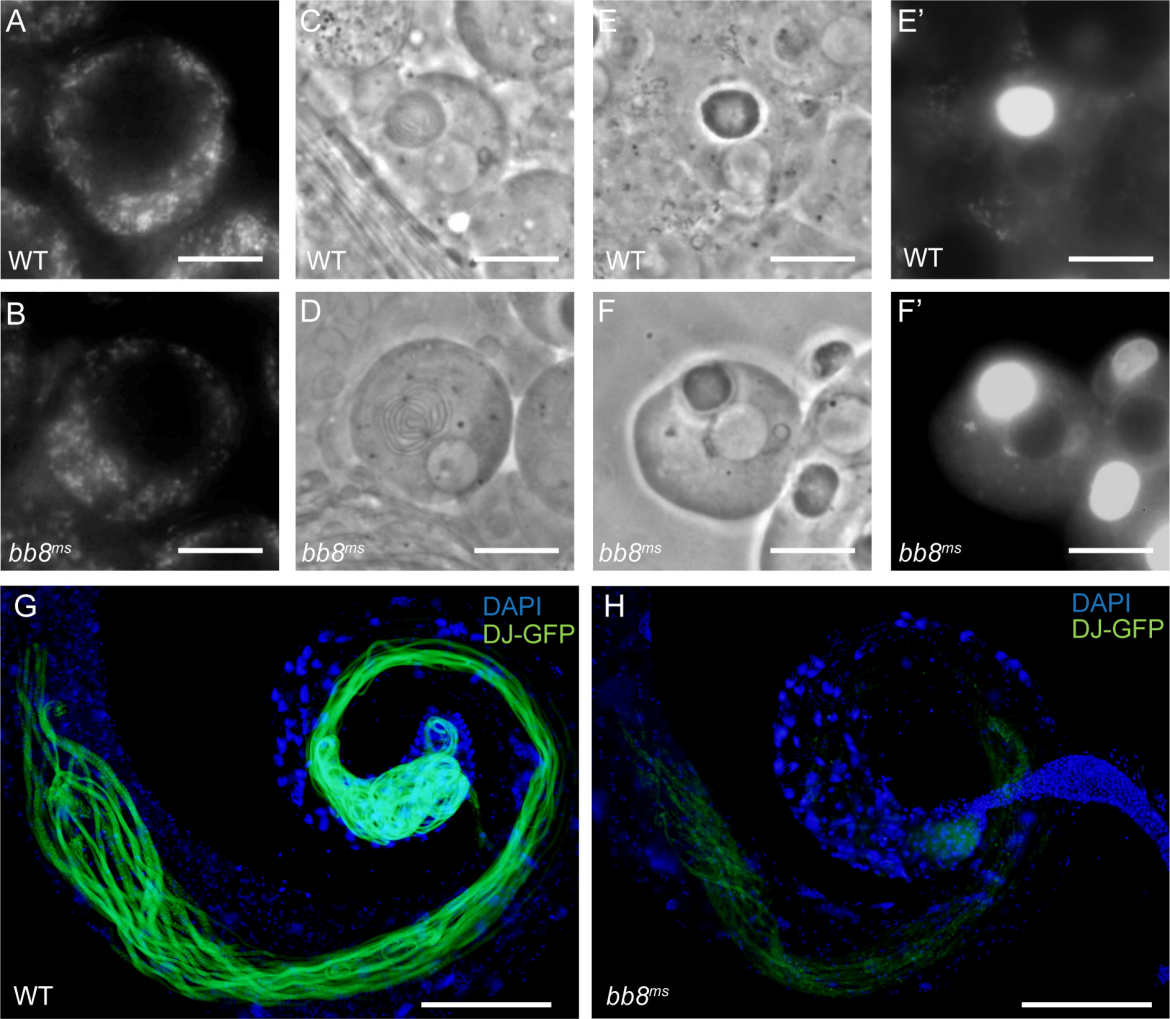
Normal mitochondrial development before spermatid elongation. (A-B) Primary spermatocytes have normal mitochondria, stained with Mitotracker in WT (A) and *bb8*^*ms*^ (B) mutants. (C-F’) Development of Nebenkern in *bb8*^*ms*^ mutant (D, F) is normal, similar to WT (C, E) by phase contrast microscopy and mitochondrial sensitive Mitotracker staining in post-meiotic onion stage spermatids (E’, F’). Scale bars: 5 μm. (G-H) Mitochondria of elongated spermatids are decorated by DJ-GFP in WT (G), but not in *bb8*^*ms*^ mutants (H). Nuclei stained with DAPI. Scale bars: 200 μm.

After meiosis, mitochondria aggregate, fuse to form the Nebenkern and elongate. The elongation of the spermatid tail is promoted by the elongation of the two mitochondrial derivatives of the spermatids. To test the development and function of mitochondria in different stages of spermatogenesis, we used phase-contrast microscopy and labelled mitochondria with the vital stain Mitotracker in dissected testes (Fig 4A–4F).

**Fig 5:**
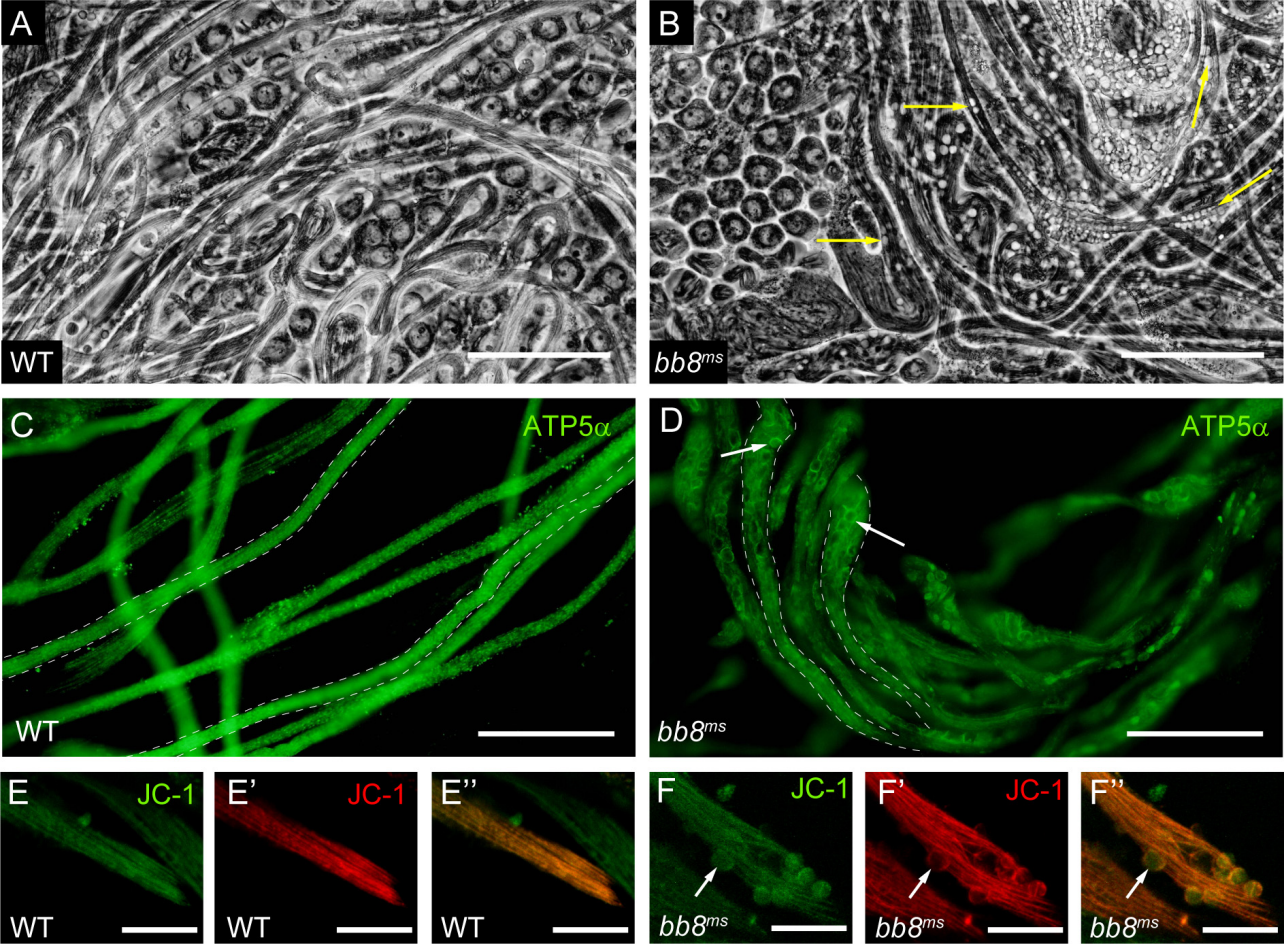
Testes of *bb8*^*ms*^ mutants show defects in post-meiotic, elongated spermatids. (A-B) Spermatids from WT (A) and *bb8*^*ms*^ (B) testis both have elongated cysts, but there are large spherical vesicles in the mutant (arrows) by phase contrast microscopy. Scale bars: 100 μm. (C, D) Mitochondria of elongated spermatids stained with ATP5α antibody in WT (C) and in *bb8*^*ms*^ (D) mutants. ATP5α positive staining of the large vesicles in the cysts are indicated by arrow. Scale bars: 50 μm. (E, F) JC-1 staining positive large vesicles (arrows) are absent from WT (E), but present in *bb8*^*ms*^ elongated cysts (F). Scale bars: 25 μm.

Primary spermatocytes show wild type morphology of mitochondria in the *bb8*^*ms*^ mutant (Fig 4A and 4B). We also observed normal organization of Nebenkern with phase contrast microscopy and with Mitotracker staining in *bb8*^*ms*^ mutant spermatids (Fig 4C–4F). Elongation defects could be the consequence of mitochondrial abnormality, so we tested mitochondria of the post-meiotic cells with a DJ-GFP transgenic line. DJ-GFP decorates the mitochondria of the elongated spermatids at the onset of spermatid individualization and mature sperm (Fig 4G) [23] [24]. In the *bb8*^*ms*^ mutant a very weak DJ-GFP signal was detectable, suggesting a mitochondrial problem in the elongating spermatids (Fig 4H). Additionally, another very striking phenotype of the mutant testes is the appearance of large, spherical vesicles inside the elongated cysts, observed using phase contrast microscopy (Fig 5A and 5B).

Both the revertant lines, generated by the precise excision of the Minos transposon and the expression of the genomic rescue construct in a *bb8*^*ms*^ mutant background, produced normal individual sperm, suggesting that the disruption of *bb8* is responsible for the large spherical vesicle formation (S2E–S2H Fig). Testes of the mutant allele of the mitochondrial iron metabolism gene *mitoferrin* (*dmfrn*^*SH115*^) also show similar vesicular structures, however their nature has not been characterized [25]. Based on the mitochondrial localization of Bb8, we decided to test these spherical objects with a mitochondria-specific, ATP5 synthase (ATP5-α), staining. We observed an ATP5 synthase signal in the large vesicles of the *bb8*^*ms*^ mutants (Fig 5C and 5D and S4A and S4B Fig). This result suggests that the lack of *bb8* function resulted in the formation of swollen mitochondria (megamitochondria) in the elongated cysts of the *bb8*^*ms*^ mutant. To test the functionality of the megamitochondria of the *bb8*^*ms*^ mutant testes, we performed a staining using JC-1, a membrane potential sensitive membrane permeable dye. JC-1 accumulates potential dependently in mitochondria and shows an emission shift from green to red in mitochondria with membrane potential [26]. We found that in the *bb8*^*ms*^ mutant, a part of the megamitochondria are still functional, based on the positive JC-1 signals (Fig 5E and 5F). We got similar results with Mitotracker staining (S4C and S4D Fig). *Big bubble* 8 is named after the presence of megamitochondria in *bb8*^*ms*^, where their diameter is ~8 μm (S4B Fig).

**Fig 6:**
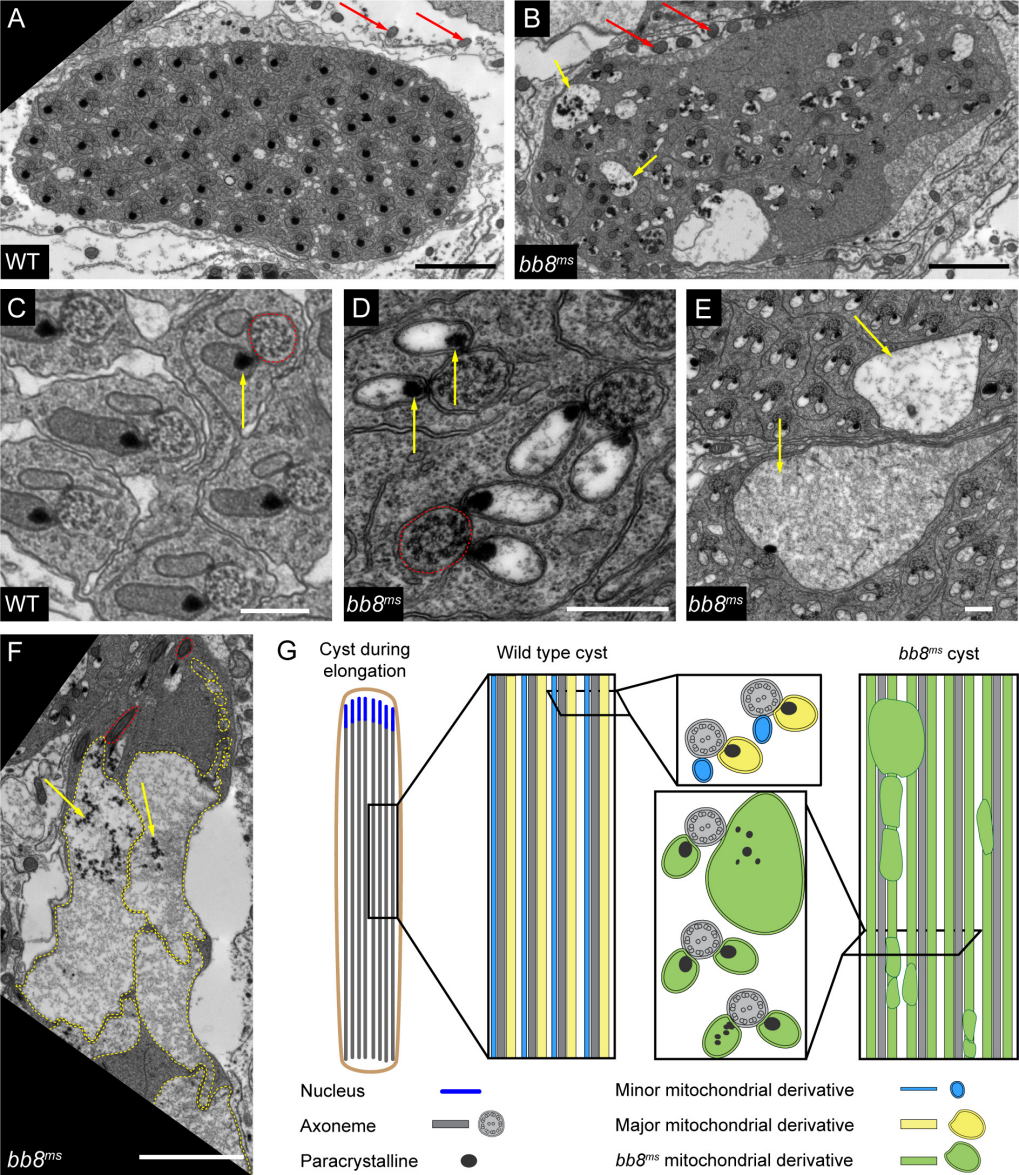
Abnormal paracrystalline formation in *bb8*^*ms*^ mutants. (A-F) Transmission electron micrographs of cross-section of testis from WT (A, C) and *bb8*^*ms*^ flies (B, D, E, F). Somatic mitochondria are indicated by red arrow (A, B). In WT preindividualized cysts (A, C), each spermatids have two mitochondrial derivatives with paracrystalline in the major derivative (yellow arrow) and an axoneme (C) (red line). *bb8*^*ms*^ pre-individualization spermatids lost their synchrony in the cyst and the paracrystalline in the mitochondrial derivative often fragmented (B, F) (yellow arrow). There are numerous swollen mitochondrial derivatives in *bb8*^*ms*^ cysts (B, E, F) (yellow arrows and yellow line). Both mitochondrial derivatives start to accumulate paracrystalline in *bb8*^*ms*^ cyst (D-F) (yellow arrow). Scale bar: 2 μm (A, B, F) 0.5 μm (C-E). (G) Schematics depicting the mitochondrial phenotypes of WT and *bb8*^*ms*^ elongating spermatids.

The lack of DJ-GFP localization and the megamitochondria formation suggest structural and/or functional problems with the post-meiotic mitochondria. At the ultrastructural level, electron microscopic examination of *bb8*^*ms*^ testes confirmed the lack of highly ordered individualized cysts and mitochondrial abnormality (Fig 6A–6F).

In wild type elongated cysts, each spermatid contains two mitochondrial derivatives parallel to the axoneme (Fig 6A and 6C). One of them becomes the large mitochondrial derivative, with paracrystalline accumulation. The other one becomes the minor mitochondrial derivative, which reduces in size and volume until individualization is completed (Fig 6A and 6C). In *bb8*^*ms*^ mutant spermatids, both of the mitochondrial derivatives are formed, but they remain similar to each other in early elongating spermatids (Fig 6B and 6D). In *bb8*^*ms*^ mutant spermatids, both mitochondrial derivatives start to accumulate paracrystalline, a hallmark of only the major mitochondrial derivative in the wild type spermatids, suggesting that the identities of the mitochondrial derivatives are disturbed. Furthermore, the structure of paracrystalline is irregular in the *bb8*^*ms*^ mutant (Fig 6B and 6F). We detected very large mitochondrial derivatives in more advanced mutant spermatids, which could correspond to the megamitochondria observed in the phase contrast and fluorescent images (Fig 5A–5F, S4A–S4D Fig). Somatic mitochondria of the mutant show normal, wild type appearance (Fig 6A and 6B).

## Discussion

During *Drosophila* spermatogenesis, synchronized spermatid individualization starts after cyst elongation, which involves dramatic morphological changes in the organelles of spermatids. Both axoneme and mitochondria elongate along the entire length of the sperm tail, where mitochondria support the elongation and offer a structural platform for microtubule reorganization [3]. These changes happen after meiosis, when transcription is very limited [16], [18]. We found that mitochondrial *bb8*, a putative glutamate dehydrogenase is expressed exclusively in the testis and the *bb8* mRNA is enriched in the post-meiotic stages of spermatogenesis. Gene duplication has resulted in testis-specific forms of many basic cellular proteins, which are specialized to perform specific tasks required for sperm formation, such as elongation and individualization of spermatids [27][28]. These proteins have a wide variety of molecular functions, including cytoskeletal proteins, protein degradation, and metabolic enzymes. More than 60% of the testis-expressed genes are over-expressed in meiotic stages, supporting the idea that they might have major roles in elongation and individualization of spermatids [18]. Nuclear-encoded mitochondrial genes are one of the most represented group of duplicated genes, where gene relocation correlates with sex-specific expression in males [28].

Recent genome analysis suggests that the specialized mitochondria of the male germline operate with a separate set of testis-specific gene products [28]. Drosophila glutamate dehydrogenases, Gdh and Bb8, may be the results of gene duplication, which might have contributed to the development of specialized mitochondrial function during spermatogenesis. Based on our sequence analysis, we showed that both *Gdh* and *bb8* are present in all of the analysed *Drosophila* species and evolved as distinct phylogenetic branches. *Gdh* is a conserved housekeeping gene with a single copy in all *Drosophila* species. *bb8* orthologues are present in all of the tested *Drosophila* species (Fig 1B and S2A Fig). *Drosophila mojavensis*, *Drosophila viridis* and *Drosophila pseudoobscura* have multiple *bb8* orthologues, however we do not have any data on their tissue-specific expression. These results suggest that the initial *bb8* duplication was not a recent event, therefore the testis-specific mitochondrial function is evolved early in the evolution of Drosophilidae.

It is well known that mutations in genes necessary for mitochondrial proliferation or fusion in spermatids result in shortened cysts [3]. In *bb8*^*ms*^ mutant, we found that the elongation of the cysts is disturbed, probably due to the striking morphological changes in the mitochondrial derivatives, and the formation of megamitochondria. We cannot exclude that the malformation of mitochondria and individualization complexes are the result of an energy deficit. According to the *Drosophila* metabolome map, glutamate and glutamate-related amino acids (proline, histidine, arginine, glutamine) are in the top 7 most abundant metabolites in the whole fly [29]. In testes, glutamine and glutamate levels are elevated, D-proline and L-histidine levels are similar and L-arginine levels are decreased compared to the whole fly. It is known that rapidly dividing cells use a lot of glutamine as an energy source [30]. In spite of the measured activity of the mitochondria in *bb8*^*ms*^ mutant testes, the lack of Bb8 expression could contribute to a failure to utilise testicular glutamine and glutamate pools in the citric acid cycle during elongation and determination of the major and minor mitochondrial derivatives of the spermatids. This could result in an energy deficit, which hinders proper mitochondrial elongation and the initiation of spermatid individualization in *bb8*^*ms*^ mutants.

Both Gdh and Bb8 are localized in the mitochondria, but somatic cells and post-meiotic spermatids have morphologically, and probably functionally, very different mitochondria [31]. This could explain why several testis-specific mitochondrial genes, such as *fzo*, *parkin*, *Hsp60* or *cyt-c-d*, have increased expression in meiotic over mitotic stages [32] [9] [8] [33] [34]. The lack of testis-specific Bb8 resulted in the formation of megamitochondria and the abnormal enrichment of paracrystalline in both mitochondrial derivatives (Fig 6G). The loss of the potential glutamate dehydrogenase activity of Bb8 could cause both a lack of alpha-ketoglutarate and/or elevation of glutamate levels. It is difficult to distinguish which of the above-mentioned molecules is responsible for the phenotype, since both are metabolic intermediates and glutamate plays a role as a signalling molecule [13]. It was demonstrated that elevation of glutamine levels by reducing selenophosphate synthetase, resulted in megamitochondria formation in *Drosophila* S2 cells [35]. Also, the manipulation of glutamate uptake with downregulation of *dmGlut*, a mitochondrial glutamate transporter, demonstrated that glutamate accumulation is a limiting step in megamitochondria formation in S2 cells [36]. The formation of megamitochondria through this pathway does not result in apoptosis, and the membrane potential of the megamitochondria is unaltered. We could imagine that the locally elevated glutamate could be responsible for megamitochondria formation in *bb8*^*ms*^ mutant (Fig 6G).

How paracrystalline formation is controlled and what paracrystalline itself is unknown. Our results suggest that glutamate/glutamine biosynthesis is important in the initiation or in the inhibition of paracrystalline formation in the mitochondrial derivatives of spermatids. Lack of Bb8 activity could induce paracrystalline formation in the small mitochondrial derivative, but it is similarly possible that the proper glutamate dehydrogenase function could contribute to the inhibition of paracrystalline formation in the minor derivative in normal spermatid development. A similar phenotype was found in the mutant of *emmenthal* and *mitoferrin* [37], [25]. In the case of *emmenthal*, there is no information about the affected gene. However it is known, that *mitoferrin* is a testis-specific mitochondrial iron transporter gene. The phenotype of *dmfrn*^*SH115*^ and *bb8*^*ms*^ mutants is remarkably similar, both in the megamitochondria formation and in the enrichment of paracrystalline in both mitochondrial derivatives. This observation is raises the possibility that glutamate biosynthesis and iron metabolism are both necessary to the normal differentiation of mitochondrial derivatives and the paracrystalline restriction to the major derivative. It is known that iron acts as a negative regulator of glutamine synthetase [38]. In *Drosophila* there are two glutamine synthetases, but none of them have testis-specific expression. It will be interesting to investigate whether iron could have a negative effect on glutamine synthesis in *Drosophila* testis.

In this study we demonstrated that the Bb8 protein contributes to the post-meiotic mitochondria elongation and restricts paracrystalline material accumulation to only one of the mitochondrial derivatives of spermatids.

## Materials and Methods

### Fly stocks and mutants

All fly stocks were maintained on standard *Drosophila* cornmeal agar medium at 25°C. Oregon-R was used as wild type control. Fly stocks used in this study were obtained from the Bloomington Drosophila Stock Center: *w*^*1118*^*;Mi{ET1}CG4434*^*MB10362*^, *w*^*1118*^*; Df(3R)Exel9012 /TM6B*, *Tb*^*1*^, *w*^*1118*^*; Df(3R)BSC619/TM6C*, *cu*^*1*^ *Sb*^*1*^, *w*^*1118*^*; noc[Sco]/SM6a*, *P{hsILMiT}2*.*4*, *w*^*1118*^*; P{DJ-GFP*.*S}AS1/CyO*. All other lines were established for this study. Individual males (30–50 per genotype) were crossed with four Oregon-R virgin females for fertility test. Five days after crossing, tubes were tested and males with no offspring considered as sterile.

### Generation of rescue and GFP reporter constructs

Genomic DNA was purified from 30 wild type flies. To make a genomic rescue construct 3097 bp PCR fragment- including 1081 bp upstream region from *bb8* (CG4434) and the full genomic region- was amplified with Phusion® High-Fidelity DNA Polymerase (Thermo Scientific). NotI-XbaI fragment was inserted into P-transposon vector, PUAST. For the Bb8-GFP-reporter construct 1081 bp long 5’ upstream region of *bb8* and the first 391 bp of *bb8* was amplified and cloned into pJET 1.2 vector (CloneJET PCR Cloning Kit, Life Technologies) and sequenced. NotI-XhoI fragment was inserted into a modified pUAST vector, where eGFP coding region was inserted in frame into XhoI-XbaI sites. Transgenic flies were generated by standard germline transformation technique. Primers used:

bb8_Resc_Rev: ACTGCGTCTAGAATGCCAATATAGGTTGTAAGTTG; bb8_Resc_Rep_Fw: ACTGCGGCGGCCGCCGAGCGTTATTGTTTACCAAG; bb8_Rep_Rev: ACTGCGCTCGAGGGGAAAATTAACCTCCACCGA; GFP_Fw: ACTGCGCTCGAGATGGTGAGCAAGGGCGAGG; GFP_Rev: GCTCTAGACTATTGTACAGCTCGTCCATGCC.

### Quantitative RT-PCR

Total RNA was purified from 30 pairs of testes, 30 heads, and 10 carcasses for each genotype. For the stage-specific purification 50 pairs of wild type testis tips and post-meiotic regions were dissected in ice cold PBS. Total RNA was purified with SV Total RNA Isolation System (Promega). For the first strand cDNA synthesis, RevertAid™ First Strand cDNA Synthesis Kit (Life Technologies) was used according to manufacturer’s instruction. Maxima SYBR Green/ROX qPCR Master Mix (Life Technologies) was used for the real time quantitative PCR reaction, according to manufacturer’s instructions. Reactions were run in triplicates in the CFX96 Real-Time PCR Detection System (Bio-Rad) with the following reaction conditions: 95°C 10 min, 50 cycles of 95°C 15 sec, 54°C 30 sec, 72°C 30 sec. *CG10252* or *rp49* specific primers was used as internal controls in the PCR reaction. Q-RT-PCR data analysis was performed with Bio-Rad CFX Manager 3.1. List of primers used in quantitative RT-PCR:

sunz_QPCR_FW: GTGTGTTCTTCAACGGAAGTCTG; sunz_QPCR_Rev: GTGAAGAATTGTTCAATGGCCAC; CG3927_QPCR_FW: GTCGGCCAAGAAAAGTAACGGAC; CG3927_QPCR_Rev: TACTTGGGAGCCCTATTCCTG; CG10252_QPCR_FW: GTCCCAATGCCTACAAGTACG; CG10252_QPCR_Rev: CCCGGAGAATTCGTCTTGTTC; GDH_QPCR_Rev: CACGTTACCGAAGCCCTGGAC; GDH_QPCR_FW: GCCTGGAGAACTTCATCAACG; bb8_QPCR_Rev: CATGAATGCCCCGATAGTCAAC; bb8_QPCR_FW: GATATCCGCCTCTGTGGTGC.

### *In situ* hybridization

cDNA was isolated from wild type testes and the 1182 bp long PCR product was used in the synthesis of sense and antisense probe with DIG RNA Labeling Kit (SP6/T7) (Roche) according to the manufacturer’s instructions. *In situ* hybridization was performed as earlier described by White-Cooper with the following differences: hybridization buffer contains additional 100 μg tRNA (Sigma) [39]. We used the sense DIG-labelled *bb8* RNA as a negative control and found no staining with it (S1D Fig). Images were taken by using Olympus BX51 microscope.

### Staining and microscopy

Testis preparation and staining were performed as earlier described by White-Cooper [40]. Mouse anti-pan polyglycylated Tubulin Antibody, clone AXO 49 (Merck) was used at a 1:5000 dilution. Mouse anti-ATP5A antibody [15H4C4] (Abcam) was used in a 1:100 dilution. Secondary antibodies Alexa Fluor 488 conjugated anti-mouse (Invitrogen) was used at a 1:400 dilution. 4',6-diamidino-2-phenylindole (DAPI) were used at 1μg/ml concentration. Texas Red®-X Phalloidin (Life Technologies) was used at a 1:250 dilution. Mitotracker Red CMXRos, (Life Technologies) was used at a 0,5 μM concentration and JC-1 (Molecular Probes) was used at 5 μg/ml concentration, both of them diluted in PBS and dissected testes were stain for 5 minutes. Samples were mounted in SlowFade® Gold antifade reagent (Life Technologies). Images were taken by using Olympus BX51 fluorescent microscope or Olympus Fluoview Fv10i Confocal microscope. Length of elongated cysts were measured by ImageJ. Electron microscopic analysis of testes were done as described in Laurinyecz et al. [41]. Images were processed with the GIMP 2.8.6.

### Bioinformatical and statistical analyses

Gdh and Bb8 orthologous protein sequences were obtained from FlyBase and OrthoDB v8 [42]. Protein sequence alignments were made by ClustalW [43]. Phylogenetic trees were constructed by using the maximum-likelihood method with MEGA 6.06 software [44]. Numbers at nodes represent the percentage bootstrap value of 1000 replicates, only values higher than 70% are shown. Length measurement data (Fig 3E and 3F) is represented as mean ± s.e.m. and were analysed for significance with Student’s *t-*test. Individualization phenotype (S3C Fig) boxplot analysis was performed with R 3.2.4 and Welch two sample t-test was used to determine significance.

## Supporting Information

S1 Fig Fertility of *bb8*^ms^ and expression of *bb8* and *Gdh* mRNA. (A) Measurement of fertility of different genotypes. (B) Ubiquitous expression of *Gdh* in different *Drosophila* tissues. Relative *Gdh* expression measured by Q-RT-PCR from WT and *bb8*^*ms*^ mutant, from isolated head, carcass and testis samples, using *rp49* as reference. Measurements were made in triplicate. (C) Relative expression of *bb8* mRNA in wild type, heterozygous, and homozygous *bb8*^*ms*^ testes using *rp49* as an internal control. Measurements were made in triplicate. (D) In wild type testis, there is no signal with the sense *bb8* DIG-RNA probe in *in situ* hybridization. Scale bar: 200 μm. (E) Isolated testis regions were used to purify mRNA for Q-RT-PCR. Scale bar: 200 μm.
(TIF)

S2 Fig Phenotypic characterization of *bb8*^*ms*^ mutant. Rescue the individualization phenotype with precise excision of the Mi{ET1}CG4434^MB10362^ (Δ*bb8*^*MB10362*^) and introducing a genomic rescue transgene into *bb8*^*ms*^ mutant. (A-H) Phase contrast microscopy of the apical region of the testis (A-D) and the part with elongated cysts (E-H) of wild type (A, E), *bb8*^*ms*^ mutant (B,F), Δ*bb8*^*MB10362*^ (C, G) and *bb8* genomic rescue lines, (P{*bb8*^*gr*^}; *bb8*^*ms*^) (D, H). (I-T) Visualization of elongation of the nuclei with DAPI staining (blue) (I-L), migrating individualization complexes (IC) (M-P) with Texas Red-X phalloidin staining (red) in elongated cysts and seminal vesicle (Q-T) in wild type (I, M, Q), *bb8*^*ms*^ mutant (J, N, R), Δ*bb8*^*MB10362*^ (K, O, S) and P{*bb8*^*gr*^}; *bb8*^*ms*^ (L, P, T). Scale bars: 50 μm
(TIF)

S3 Fig Individualization complexes in *bb8*^*ms*^ mutant (A-B) Individualization complex formation visualized with Texas Red-X phalloidin in the wild type (A) and *bb8*^*ms*^ mutant (B) cysts. (C) Quantification of the percentage of late elongating cysts at various phenotypes in WT and *bb8*^*ms*^ mutant testes. Statistical significance was determined by Welch two sample t-test. n represents the number of analysed cysts per genotype.
(TIF)

S4 Fig Megamitochondria in *bb8*^*ms*^ mutant. (A, B) ATP5α staining (green) in WT spermatids (A). No abnormality observed in mitochondria of the round spermatids (arrow), but mitochondria are swollen in elongated cysts in *bb8*^*ms*^ (arrowhead) (B). Nuclei are stained with DAPI. (C, D) Swollen mitochondria present (arrowhead) with Mitotracker (red) staining in *bb8*^*ms*^ spermatids. Scale bars: 10 μm
(TIF)
