# Supplementary figures and images for: Testis-Specific Bb8 Is Essential in the Development of Spermatid Mitochondria
Source: PLoS One. 2016 Aug 16;11(8):e0161289. doi: 10.1371/journal.pone.0161289 (PMC4986964; doi:10.1371/journal.pone.0161289)

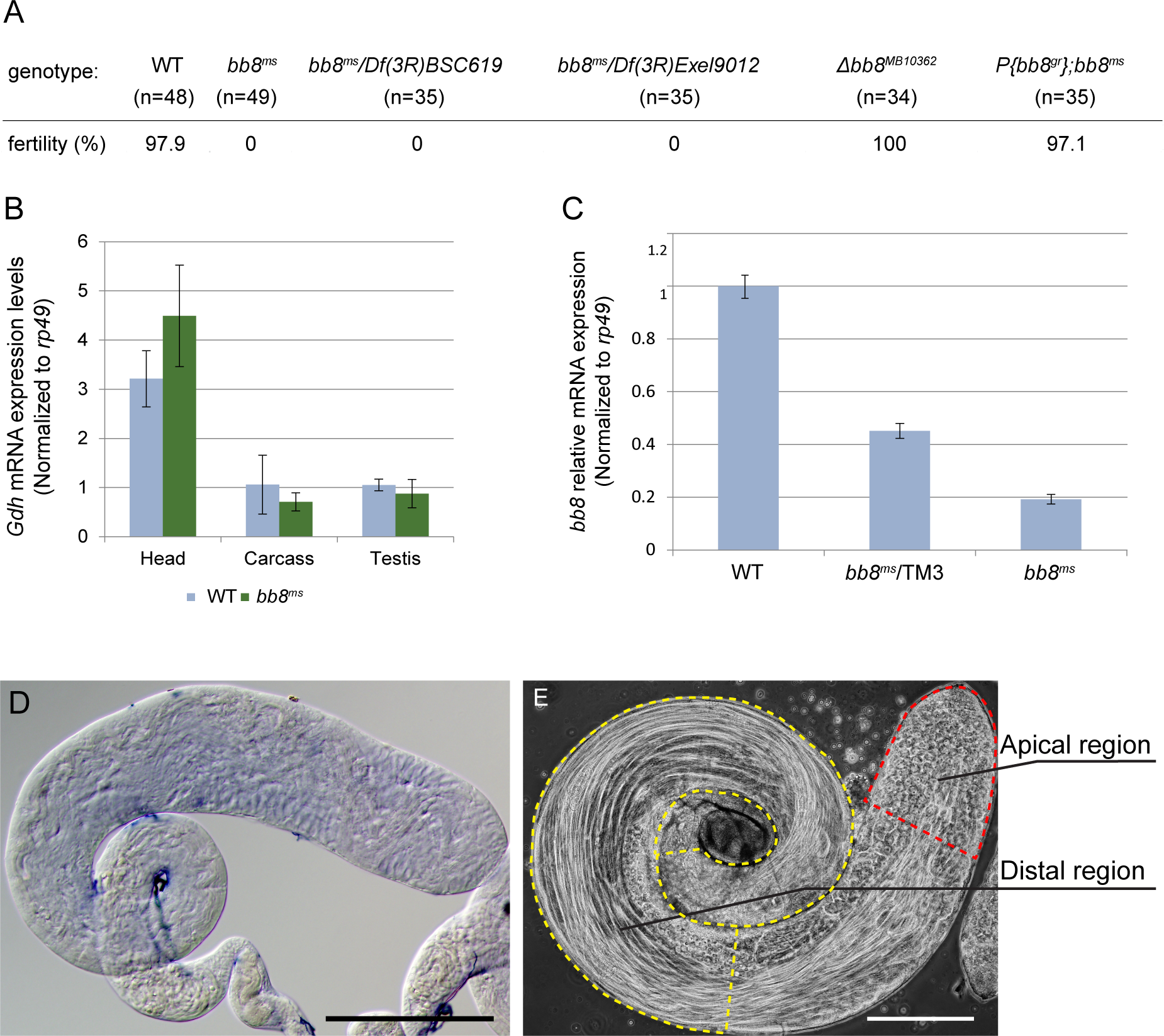

Supplement: S1 Fig — (A) Measurement of fertility of different genotypes. (B) Ubiquitous expression of Gdh in different Drosophila tissues. Relative Gdh expression measured by Q-RT-PCR from WT and bb8ms mutant, from isolated head, carcass and testis samples, using rp49 as reference. Measurements were made in triplicate. (C) Relative expression of bb8 mRNA in wild type, heterozygous, and homozygous bb8ms testes using rp49 as an internal control. Measurements were made in triplicate. (D) In wild type testis, there is no signal with the sense bb8 DIG-RNA probe in in situ hybridization. Scale bar: 200 μm. (E) Isolated testis regions were used to purify mRNA for Q-RT-PCR. Scale bar: 200 μm. (TIF) [file pone.0161289.s001.tif]

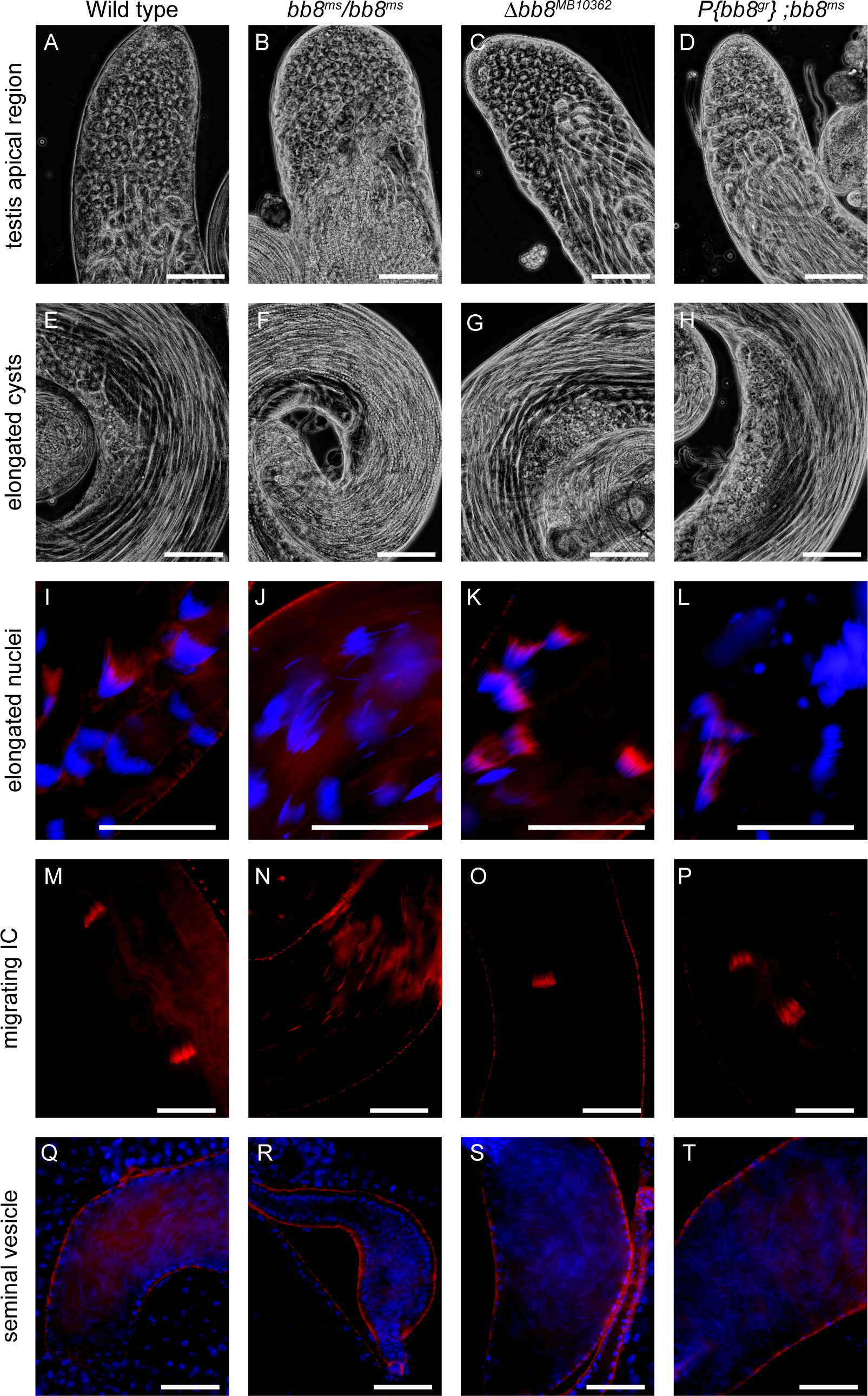

Supplement: S2 Fig — Rescue the individualization phenotype with precise excision of the Mi{ET1}CG4434MB10362 (Δbb8MB10362) and introducing a genomic rescue transgene into bb8ms mutant. (A-H) Phase contrast microscopy of the apical region of the testis (A-D) and the part with elongated cysts (E-H) of wild type (A, E), bb8ms mutant (B,F), Δbb8MB10362 (C, G) and bb8 genomic rescue lines, (P{bb8gr}; bb8ms) (D, H). (I-T) Visualization of elongation of the nuclei with DAPI staining (blue) (I-L), migrating individualization complexes (IC) (M-P) with Texas Red-X phalloidin staining (red) in elongated cysts and seminal vesicle (Q-T) in wild type (I, M, Q), bb8ms mutant (J, N, R), Δbb8MB10362 (K, O, S) and P{bb8gr}; bb8ms (L, P, T). Scale bars: 50 μm (TIF) [file pone.0161289.s002.tif]

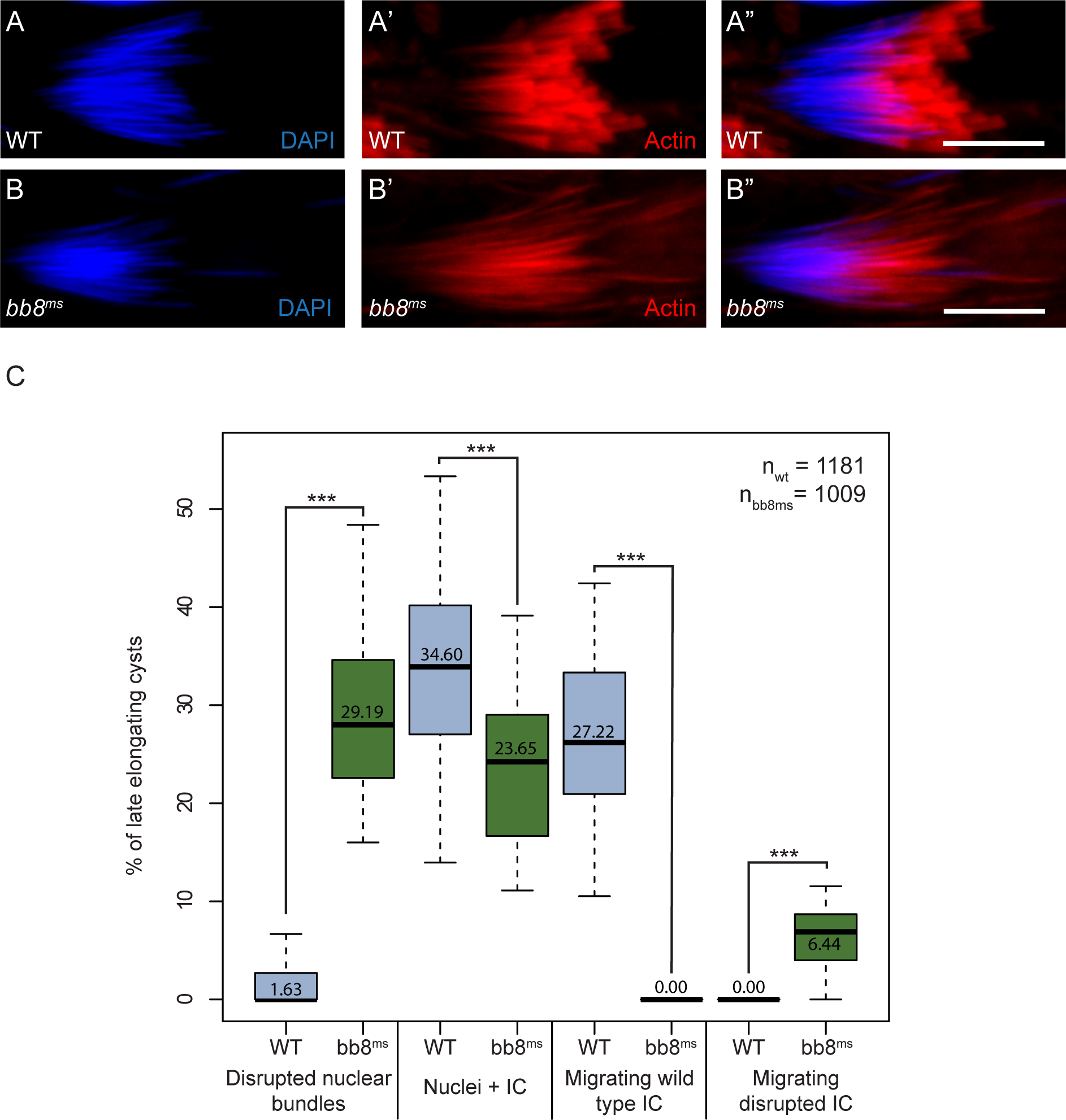

Supplement: S3 Fig — (A-B) Individualization complex formation visualized with Texas Red-X phalloidin in the wild type (A) and bb8ms mutant (B) cysts. (C) Quantification of the percentage of late elongating cysts at various phenotypes in WT and bb8ms mutant testes. Statistical significance was determined by Welch two sample t-test. n represents the number of analysed cysts per genotype. (TIF) [file pone.0161289.s003.tif]

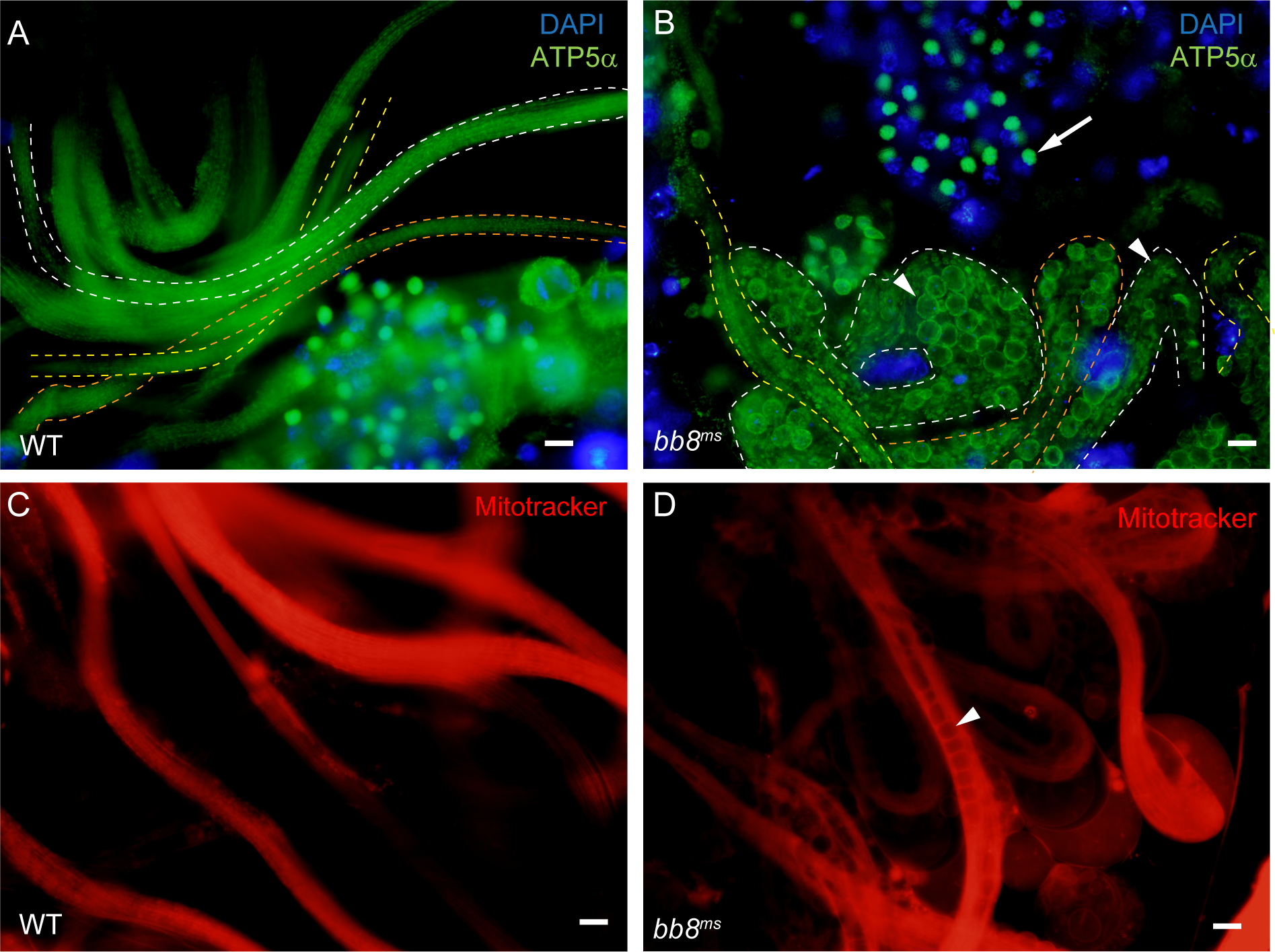

Supplement: S4 Fig — (A, B) ATP5α staining (green) in WT spermatids (A). No abnormality observed in mitochondria of the round spermatids (arrow), but mitochondria are swollen in elongated cysts in bb8ms (arrowhead) (B). Nuclei are stained with DAPI. (C, D) Swollen mitochondria present (arrowhead) with Mitotracker (red) staining in bb8ms spermatids. Scale bars: 10 μm (TIF) [file pone.0161289.s004.tif]
